# Spontaneous rupture of superficial femoral artery aneurysm: case report

**DOI:** 10.1590/1677-5449.011318

**Published:** 2019-03-06

**Authors:** Marcio Miyamotto, João Márcio dos Santos Biscardi, Cristina Detoni Trentin, Rafael Malucelli Machado, Bruna Zimmerman Angelo, Danielle Côrrea de Andrade, Cintia Lopes Raymundo, Bruno Moraes Ribas

**Affiliations:** 1 Pontifícia Universidade Católica do Paraná – PUC-PR, Hospital Universitário Cajuru – HUC, Serviço de Cirurgia Vascular e Endovascular, Curitiba, PR, Brasil.; 2 Instituto VESSEL de Aperfeiçoamento Endovascular, Curitiba, PR, Brasil.; 3 Hospital Nossa Senhora das Graças – HNSG, Serviço de Cirurgia Vascular e Endovascular Elias Abrão, Curitiba, PR, Brasil.; 4 Liga Acadêmica de Medicina Vascular – LAMEV, Curitiba, PR, Brasil.

**Keywords:** superficial femoral artery, aneurysm, arterial bypass

## Abstract

Isolated true aneurysms of the superficial femoral artery (SFA) are rare, accounting for 0.5% of peripheral aneurysms. The literature up to 2012 contains reports of just 103 patients with isolated SFA aneurysms. The main complications are thrombosis, distal embolization, and rupture, which is the most common of the three. The authors report the case of a 55-year-old male patient admitted to the emergency service with pain and a pulsatile mass in the left thigh, subsequently confirmed as rupture of an SFA aneurysm. The patient underwent open aneurysm repair with ligature and revascularization with a reversed saphenous vein bypass.

## INTRODUCTION

 True aneurysms of the femoral arteries generally involve the common femoral artery (CFA) or present as a proximal continuation of aneurysmal disease of the popliteal artery. Pseudoaneurysms of the femoral arteries, which have become more frequent because of greater use of percutaneous procedures, are also most common in the CFA. However, true isolated aneurysms of the superficial femoral artery (SFA) are rare, accounting for approximately 1% of all aneurysms involving the SFA – including false aneurysms –and 0.5% of true peripheral aneurysms. 1 ^,^ 2 In the literature up to 2012, there are reports of 103 cases of true isolated aneurysms of the SFA worldwide, among which the distal third was most frequently involved. 3

 Complications associated with SFA aneurysms include thrombosis, distal embolization, and rupture, the last of which is the most frequent. Although they are associated with lower rates of complications than other peripheral aneurysms, such as popliteal artery aneurysms, SFA aneurysms should be diagnosed and electively repaired in order to prevent complications. 2 ^,^ 4 ^,^ 5

 The objective of this study is to describe treatment of a rare case of rupture of a true aneurysm of the mid third of the SFA and provide a brief review of the literature on the subject. 

## CASE DESCRIPTION

**Figure 1:**
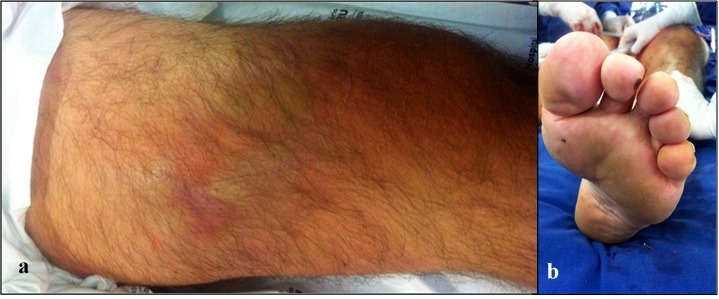
Swelling in the left mid thigh (a) and signs of distal microembolization (b).

 The patient was a 55-year-old male entrepreneur, who had been experiencing pain of moderate intensity in the mid third of his left thigh for approximately 6 days. He sought emergency care at a hospital in response to a sudden increase in the intensity of the pain combined with swelling at the site of pain. He had a history of smoking equating to approximately 37 pack years. He did not have any other comorbidities. On physical examination he was slightly pale, with tachycardia (120 bpm) and blood pressure at 100 x 70 mmHg. Physical examination by segments was unremarkable for the head and neck, thorax, and abdomen. Vascular examination of the right lower limb found normal auscultation and visual inspection results, with all pulses present and normal. The left lower limb was well-perfused, but there was ecchymosis and a pulsatile swelling between the mid and distal thirds of the thigh, in the anteromedial region ( Figure 1 a). Inspection of the left foot also revealed signs of distal microembolization ( Figure 1 b) and popliteal and distal pulses were absent. 

**Figure 2:**
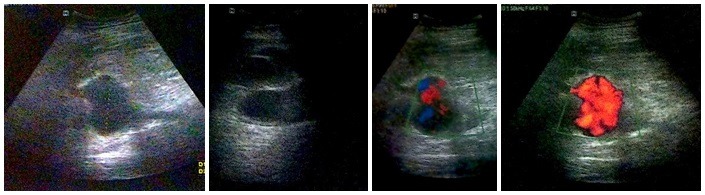
Ultrasonographic appearance of the femoral artery aneurysm.

 Ultrasonography of the left thigh showed an aneurysmal dilatation of the SFA measuring 5.8 x 5.3 cm and with associated mural thrombi and perivascular accumulations compatible with a ruptured aneurysm ( Figure 2 ). Since angiotomography was not available at the service providing care, the decision was taken to perform emergency surgical treatment. 

**Figure 3:**
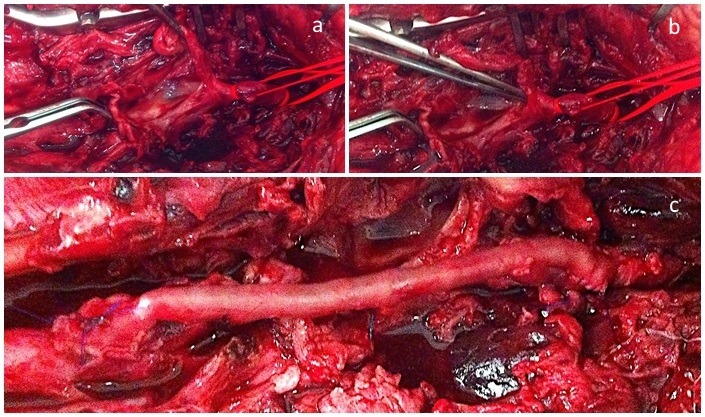
Intraoperative images showing the ruptured femoral artery aneurysm, dissected with proximal and distal repair (a, b); Interposition of the reversed great saphenous vein (c).

 During the procedure, extensive hematoma was observed involving subcutaneous and muscle tissues in the anteromedial region of the thigh. Once this had been removed, the ruptured SFA aneurysm could be seen ( Figures 3 3b). There were no obvious signs of active infection. Proximal and distal ligatures were performed and then the aneurysm was resected and samples collected for anatomopathological and microbiological analyses. Revascularization of the limb was then accomplished by interposition of the contralateral great saphenous vein in reverse, with end-to-side anastomosis – taking into consideration the diameter of the femoral artery and the significant destruction of its walls, as illustrated in Figure 3 c. The contralateral saphenous vein was used both because of the probability of associated damage to deep veins in the limb involved in rupture and because of the greater likelihood of injury during dissection, due to anatomic distortions. There were no intercurrent conditions during the procedure 

 The patient was prescribed prolonged, wide-spectrum antibiotic therapy until the results of the microbiological culture of the aneurysmal fragment were available, showing no evidence of growth of microorganisms. The anatomopathological analysis found true aneurysmal walls, with no specific abnormalities. Supplementary imaging exams did not identify any additional aneurysms or any evidence of valve vegetations suggestive of endocarditis. 

 At 1-month follow-up, the patient had palpable distal pulses and was free from pain or other problems. 

## DISCUSSION

 Aneurysms involving the SFA and considered atherosclerotic generally present as a distal extension of an aneurysm of the CFA or a proximal continuation of an aneurysm of the popliteal artery, via the adductor canal. Isolated aneurysms of the SFA are often secondary to traumas (pseudoaneurysms) or to conditions related to inflammatory and infectious processes, or connective tissue diseases. However, isolated atherosclerotic aneurysms of the SFA are rare and account for 15 to 25% of true aneurysms involving this region. 3 ^,^ 6 ^,^ 7

 The most common location of SFA aneurysms is the distal third of the artery (59%) and they tend to be focal, rarely involving a long segment of the artery. 3 In the current report, the aneurysm was in the mid third of the SFA, increasing the rarity of this case. 

 Aneurysms of the SFA can be associated with aortoiliac aneurysms, in up to 69% of cases, and with aneurysms of the popliteal artery or CFA, in up to 54% of cases. 6 Bilateral aneurysms of the SFA can occur in up to 38% of cases. 3 A complete investigation for aneurysms in other sites should be conducted at the time of diagnosis and also at later follow-up, in order to detect new aneurysms. In the case described here, imaging exams were used to screen for associated aneurysms, as recommended in the literature, and no further abnormalities were identified. 

 Additionally, aneurysms of the SFA predominantly affect men (85%) and this disparity can be even greater when popliteal artery aneurysms are analyzed (97%). 8 The fact that the patient in this report was male reinforces this epidemiological characteristic of SFA aneurysms. 

 True isolated aneurysms of the SFA tend to be symptomatic at the time of diagnosis (35% of cases) in a greater proportion than aneurysms of the CFA or popliteal artery (7% of cases). This observation may be because of the difficulty of observing and palpating this type of aneurysm, making early identification and elective surgical repair less likely. Recognition of an SFA aneurysm by palpation is unlikely, even in lean patients, because of the depth of the artery’s course and the protection provided by the musculature of the thigh. 6 Additionally, aneurysms of the SFA tend to have larger diameters at the time of diagnosis (a mean of 5.4 cm). 3 ^,^ 7 ^,^ 8 In line with the literature, the present case involved rupture of an SFA aneurysm of considerable size (5.8 x 5.3 cm). 

 With regard to symptoms, the literature identifies rupture (26 to 34%) as the most common presentation of SFA aneurysms, a higher rate than with aneurysms of the popliteal artery (3%). 6 ^,^ 9 Ischemic symptoms are also a common initial presentation in these patients (22%). 6 The patient in this study had rupture as initial presentation, which is also in line with what is reported in the literature. 

 Treatment of isolated aneurysms of the SFA follows the same principles applicable to repair of aneurysms in other anatomic sites: removal of the source of embolism, prevention or treatment of rupture, elimination of mass effects, and restoration of perfusion to the limbs. 8 Surgical repair is indicated for all symptomatic patients. With regard to asymptomatic patients, there is still no consensus in the literature on the aneurysm diameter at which the probability of complications is great enough to justify elective treatment, although some authors suggest that aneurysms larger than 20 to 25 mm are candidates for surgical intervention. 3 ^,^ 8 The most common treatment is aneurysmectomy with reconstruction by interposition of a prosthetic graft, which is a technique that affords anatomopathological analysis of the aneurysm to correctly identify the underlying etiology. In cases with acute ischemia and a need to access the distal popliteal artery for selective thrombectomy, the aneurysm can be excluded by constructing a femoropopliteal bypass, preferably involving use of an autologous great saphenous vein. Endovascular treatment has only been described in three patients with isolated SFA aneurysms, but it may be an option for patients with contraindications to open repair. 10 ^,^ 11 Even when ruptured SFA aneurysms are repaired during emergency surgery, the literature still reports good results, with early mortality of 4% and limb salvage rates estimated at 88% at 5 years. 3 In the case described here, an aneurysmectomy was performed with immediate reconstruction of the course of the artery using interposition of an autologous saphenous vein graft, harvested from the contralateral limb. The choice of an open technique was based both on the existence of many reports of its efficacy and on the fact that it enables confirmation of the etiology of the aneurysm. 

## CONCLUSIONS

 This report describes management of a male, 55-year-old patient, admitted after sudden exacerbation of pain of initially moderate intensity in the mid third of the left thigh, approximately 6 days after onset. Physical examination and ultrasonography confirmed the suspicion of a ruptured isolated SFA aneurysm. Since the aneurysm was symptomatic, aneurysmectomy was performed, followed by interposition of an autologous saphenous vein graft from the contralateral limb, in accordance with recommendations in the literature, with no intercurrent conditions – confirming the feasibility of open repair. 

## References

[1] Oliveira AF, Oliveira H (2009). Aneurisma de artéria femoral superficial roto: relato de caso e revisão de literatura. J Vasc Bras.

[2] Arendt AL, Amaral RM, Vieira MS, Ribeiro RN, Argenta R (2013). Aneurisma verdadeiro roto de arteria femoral superficial. J Vasc Bras.

[3] Perini P, Jean-Baptiste E, Vezzosi M (2014). Surgical management of isolated superficial femoral artery degenerative aneurysms. J Vasc Surg.

[4] Cronenwett JL, Johnston KW (2014). Rutherford’s vascular surgery..

[5] Papadoulas S, Skroubis G, Marangos MN, Kakkos SK, Tsolakis JA (2000). Ruptured aneurysms of superficial femoral artery. Eur J Vasc Endovasc Surg.

[6] Jarrett F, Makaroun MS, Rhee RY, Bertges DJ (2002). Superficial femoral artery aneurysms: an unusual entity?. J Vasc Surg.

[7] Siani A, Flaishman I, Napoli F, Schioppa A, Zaccaria A (2005). Rupture of an isolated true superficial femoral artery aneurysm: case report. G Chir.

[8] Leon LR, Taylor Z, Psalms SB, Mills JL (2008). Degenerative aneurysms of the superficial femoral artery. Eur J Vasc Endovasc Surg.

[9] Piffaretti G, Mariscalco G, Tozzi M, Rivolta N, Annoni M, Castelli P (2011). Twenty-year experience of femoral artery aneurysms. J Vasc Surg.

[10] Diethrich EB, Papazoglou K (1995). Endoluminal grafting for aneurysmal andocclusive disease in the superficial femoral artery: early experience. J Endovasc Surg.

[11] Troitskiĭ AV, Bobrovskaia AN, Orekhov P (2005). Successful percutaneous endovascular treatment of a ruptured femoral aneurysm. Angiol Sosud Khir.

