# Supplementary material for: Spontaneous rupture of superficial femoral artery aneurysm: case report
Source: J Vasc Bras. 2019 Mar 6;18:e20180113. doi: 10.1590/1677-5449.011318 (PMC6579521; doi:10.1590/1677-5449.011318)
Supplement: Translation

# Translation: Ruptura espontânea de aneurisma do segmento médio da artéria femoral superficial: relato de caso

**Authors:** Marcio Miyamotto, João Márcio dos Santos Biscardi, Cristina Detoni Trentin, Rafael Malucelli Machado, Bruna Zimmerman Angelo, Danielle Côrrea de Andrade, Cintia Lopes Raymundo, Bruno Moraes Ribas

**Affiliations:** 1 Pontifícia Universidade Católica do Paraná – PUC-PR, Hospital Universitário Cajuru – HUC, Serviço de Cirurgia Vascular e Endovascular, Curitiba, PR, Brasil.; 2 Instituto VESSEL de Aperfeiçoamento Endovascular, Curitiba, PR, Brasil.; 3 Hospital Nossa Senhora das Graças – HNSG, Serviço de Cirurgia Vascular e Endovascular Elias Abrão, Curitiba, PR, Brasil.; 4 Liga Acadêmica de Medicina Vascular – LAMEV, Curitiba, PR, Brasil.

**Keywords:** artéria femoral superficial, aneurisma, reconstrução arterial

## Abstract

Aneurismas verdadeiros isolados da artéria femoral superficial (AFS) são raros, representando 0,5% dos aneurismas periféricos. Até 2012, existiam relatos na literatura de apenas 103 pacientes com aneurismas verdadeiros isolados da AFS. As principais complicações associadas são: trombose, embolização distal e rotura, sendo a última a mais comum. Os autores relatam o caso de um paciente masculino, de 55 anos, atendido emergencialmente com dor e massa pulsátil em coxa esquerda, condição posteriormente diagnosticada como ruptura de aneurisma da AFS. O paciente foi submetido à correção cirúrgica emergencial com ligadura do aneurisma e revascularização com veia safena magna reversa, com evolução satisfatória.

## INTRODUÇÃO

 Aneurismas verdadeiros no território femoral habitualmente acometem a artéria femoral comum (AFC) ou apresentam-se como uma extensão proximal da doença aneurismática da artéria poplítea. Os pseudoaneurismas em território femoral, que têm ocorrido com maior frequência em função da recente expansão dos procedimentos percutâneos, também se desenvolvem mais frequentemente na AFC. Entretanto, os aneurismas verdadeiros e isolados da artéria femoral superficial (AFS) são raros, representando aproximadamente 1% de todos os aneurismas que acometem a AFS – incluindo os falsos aneurismas – e 0,5% dos aneurismas periféricos verdadeiros 1 ^,^ 2 . Até 2012, existiam, na literatura, relatos de 103 casos de aneurismas verdadeiros e isolados em AFS em todo o mundo, sendo o terço distal o mais frequentemente acometido 3 . 

 As complicações associadas aos aneurismas da AFS incluem a trombose, a embolização distal e a rotura, sendo esta observada com mais frequência. Embora associados a incidências menores de complicações em relação a outros aneurismas periféricos, como o de artéria poplítea, os aneurismas em AFS devem ser diagnosticados e corrigidos eletivamente de modo a prevenir complicações 2 ^,^ 4 ^,^ 5 . 

 O presente estudo tem como objetivos relatar o tratamento de um caso raro de rotura de um aneurisma verdadeiro do segmento médio da AFS, bem como promover uma breve revisão da literatura sobre o tema. 

## DESCRIÇÃO DO CASO

 Paciente do sexo masculino, 55 anos de idade, empresário, com quadro de dor de intensidade moderada em terço médio de coxa esquerda há aproximadamente 6 dias. Procurou atendimento hospitalar de emergência quando houve aumento súbito da intensidade da dor, acompanhado de aumento de volume no mesmo local. Tem histórico de tabagismo de aproximadamente 37 anos-maço. Não apresentava outras comorbidades. Ao exame físico, apresentava-se discretamente hipocorado, taquicárdico (120 bpm) e com pressão arterial de 100 x 70 mmHg. O exame físico segmentar de cabeça e pescoço, tórax e abdome não evidenciava anormalidades. O exame vascular do membro inferior direito revelou-se normal quanto à inspeção e ausculta, estando todos os pulsos palpáveis e sem alterações. O membro inferior esquerdo apresentava-se bem perfundido, porém, com a presença de equimose e abaulamento pulsátil entre os terços médio e distal da coxa, na região anteromedial ( Figura 1 a). Apresentava também sinais de microembolização distal na inspeção do pé esquerdo ( Figura 1 b), bem como ausência de pulsos poplíteo e distais. 

**Figura 1:** Aumento de volume em região medial de coxa esquerda (a) e sinais de microembolização distal (b).

 A ultrassonografia da coxa esquerda revelou uma dilatação aneurismática em AFS de dimensões de 5,8 x 5,3 cm, com trombos murais associados e coleção perivascular compatível com ruptura de aneurisma ( Figura 2 ). Em função da indisponibilidade da angiotomografia no atendimento inicial, optou-se pela realização do tratamento cirúrgico em caráter de emergência. 

**Figura 2:** Aspectos ultrassonográficos do aneurisma da artéria femoral.

 Durante o procedimento, identificou-se um hematoma extenso junto aos tecidos subcutâneo e muscular na região anteromedial da coxa. Uma vez retirado, visualizou-se o aneurisma roto da AFS ( Figuras 3 3b). Não havia sinais aparentes de infecção ativa. Realizaram-se, então, ligaduras proximal e distal, posteriormente ocorrendo a ressecção do aneurisma e a preparação de amostras para as análises anatomopatológica e microbiológica. Em seguida, realizou-se a revascularização do membro por meio da interposição de veia safena magna reversa retirada do membro contralateral, com anastomose de modo término-lateral – considerando-se o diâmetro da artéria femoral e também a importante destruição de suas bordas, como ilustra a Figura 3 c. A veia safena contralateral foi utilizada não somente devido à probabilidade de lesão associada das veias profundas no membro afetado pela rotura, como também pela maior possibilidade de lesão durante a dissecção por distorções anatômicas. Não houve intercorrências durante o procedimento 

**Figura 3:** Aspectos cirúrgicos demonstrando o aneurisma da artéria femoral roto e dissecado com reparo proximal e distal (a, b); Interposição de veia safena magna reversa (c).

 O paciente foi mantido sob antibioticoterapia prolongada de amplo espectro até o resultado da cultura microbiológica do fragmento aneurismático, o qual não evidenciou o crescimento de micro-organismos. A análise anatomopatológica revelou paredes de aneurisma verdadeiro, sem alterações específicas. Quanto aos exames de imagem complementares, não foram identificados outros aneurismas associados ou qualquer evidência de vegetação valvar sugestiva de endocardite. 

 No acompanhamento pós-operatório de um mês, o paciente apresentava os pulsos distais palpáveis, negando dor e demais alterações. 

## DISCUSSÃO

 Os aneurismas ditos ateroscleróticos que acometem a AFS geralmente apresentam-se como uma extensão distal de aneurismas da AFC ou como uma continuidade proximal de aneurismas de poplítea pelo canal dos adutores. Aneurismas isolados da AFS são comumente secundários a traumas (pseudoaneurismas) ou a condições associadas a processos inflamatórios e infecciosos, além de doenças do tecido conjuntivo. Entretanto, os aneurismas ateroscleróticos isolados da AFS são raros e correspondem a cerca de 15 a 25% dos aneurismas verdadeiros que acometem esse território 3 ^,^ 6 ^,^ 7 . 

 A localização mais frequente dos aneurismas de AFS é em terço distal da artéria (59%), e eles tendem a ser focais, raramente envolvendo um segmento longo da artéria 3 . No presente relato, o aneurisma situava-se no terço médio da AFS, o que reforça a raridade do quadro. 

 Aneurismas da AFS podem associar-se a aneurismas em território aorto-ilíaco em até 69% dos casos e a aneurismas da artéria poplítea e da AFC em até 54% dos casos 6 . Aneurismas bilaterais da AFS podem ocorrer em até 38% dos casos 3 . A investigação completa da presença de aneurismas em outras localizações deve ser realizada na ocasião do diagnóstico, bem como durante o seguimento tardio, a fim de detectar o aparecimento de novos aneurismas. No caso em estudo, realizou-se o rastreamento de aneurismas associados por meio de exames complementares de imagem, como preconiza a literatura, e não foram identificadas demais anormalidades. 

 Ademais, aneurismas da AFS predominam no sexo masculino (85%), sendo que essa disparidade pode ser ainda maior quando analisados os aneurismas da artéria poplítea (97%) 8 . O fato de o paciente do presente relato ser do sexo masculino reforça essa característica epidemiológica dos aneurismas de AFS. 

 Aneurismas verdadeiros e isolados da AFS comumente apresentam-se sintomáticos no momento do diagnóstico (35% dos casos), uma proporção superior à dos aneurismas de AFC ou de artéria poplítea (7% dos casos). Essa observação pode ser explicada pela dificuldade de visualização e palpação desse tipo de aneurisma, desfavorecendo a identificação precoce e a correção cirúrgica eletiva. Mesmo em indivíduos magros, o reconhecimento do aneurisma de AFS por palpação é dificultado em função da profundidade do trajeto arterial e da proteção conferida pela musculatura da região da coxa 6 . Além do mais, aneurismas em AFS tendem a apresentar diâmetros maiores no momento de seu diagnóstico (5,4 cm em média) 3 ^,^ 7 ^,^ 8 . Em concordância com a literatura, o presente relato envolveu a rotura de um aneurisma de AFS de dimensões importantes (5,8 x 5,3 cm). 

 Quanto aos sintomas, a literatura indica a ruptura (26 a 34%) como apresentação mais comum do aneurisma de AFS, que é mais frequente do que nos aneurismas da artéria poplítea (3%) 6 ^,^ 9 . Sintomas isquêmicos também são comuns (22%) como apresentação inicial nesses pacientes 6 . O paciente do presente estudo teve rotura como apresentação inicial, o que também está em conformidade com o exposto pela literatura. 

 O tratamento de aneurismas isolados da AFS segue os mesmos princípios aplicáveis ​​à reparação de aneurismas em outros sítios anatômicos: remoção da fonte embólica, prevenção ou tratamento da ruptura, eliminação dos efeitos de massa e restauração da perfusão dos membros 8 . Indica-se a correção cirúrgica a todos os pacientes sintomáticos. Quanto aos assintomáticos, ainda inexiste consenso na literatura no que diz respeito ao diâmetro do aneurisma, no qual a alta probabilidade de complicações justifica a realização do tratamento eletivo, embora alguns autores sugiram que aneurismas maiores que 20 a 25 mm devam sofrer intervenção cirúrgica 3 ^,^ 8 . O tratamento mais comumente realizado é a aneurismectomia com reconstrução por meio de interposição de enxerto protético, técnica que possibilita a análise anatomopatológica do aneurisma de modo a identificar corretamente a etiologia subjacente. Em casos de isquemia aguda e necessidade de acesso à artéria poplítea distal para trombectomia seletiva, pode-se excluir o aneurisma por meio de uma derivação femoropoplítea, preferencialmente envolvendo a utilização de veia safena magna autóloga. O tratamento endovascular foi relatado em apenas três pacientes com aneurismas isolados da AFS e pode representar uma alternativa a pacientes com contraindicações ao reparo aberto 10 ^,^ 11 . Ainda que os aneurismas rotos da AFS sejam corrigidos cirurgicamente em caráter emergencial, a literatura aponta bons resultados, que envolvem mortalidade precoce de 4% e taxa de salvamento de membro estimada em 88% em 5 anos 3 . No presente relato, realizaram-se a aneurismectomia e a imediata reconstrução do trajeto arterial, que envolveu a interposição de um enxerto de veia safena autóloga de membro contralateral. A opção pela técnica aberta embasou-se não somente na existência de vários relatos de eficácia, como também na viabilização da confirmação etiológica do aneurisma do paciente em estudo. 

## CONCLUSÃO

 O presente trabalho ilustra o manejo de um paciente masculino, de 55 anos, admitido com aumento súbito de intensidade de uma dor inicialmente moderada em terço médio de coxa esquerda e que já existia há aproximadamente 6 dias. O exame físico e a ultrassonografia confirmaram a suspeita de rotura de aneurisma isolado de AFS. Por se tratar de um aneurisma sintomático, realizou-se a aneurismectomia associada à interposição de enxerto de veia safena autóloga de membro contralateral, conforme recomendações da literatura, e não houve intercorrências – corroborando a exequibilidade do reparo aberto.
